# Editorial Correspondence

**Published:** 1877-10

**Authors:** Julian T. Moore

**Affiliations:** Cairo, Ga.


CAIRO, GA., October 8, 1877.

*Editor Atlanta Medical and Surgical Journal:*

Having seen the case of a child crying in utero published in your valuable JOURNAL, I concluded that I would send you a case equally as interesting and novel—a child born with fractured leg.

Several months since, Dr. W., of this place, requested me to assist him in reducing the fractured leg of a child. When we arrived at the house of the patient's parents, I found a child three days old with compound comminuted fracture of the tibia. The bone was broken transversely across, beginning at the middle and passing upward and outward. The fractured end had passed out, making a wound about the size of a five cent piece. When I saw it the bone had been retracted, inverting the skin, which I suppose had become attached to a spicula of bone, and the wound had healed, leaving an indentation about the size of a large pea and one-fourth of an inch deep. Both ends of the fibula were dislocated. I proposed to Dr. W. that we would cut down upon the bone and remove the portion of skin between the fractured ends, telling him that I thought setting the bone, without clearing the ends, would result in a total failure, as it was impossible for the fracture to knit together with a foreign substance intervening. He disagreed, and it being his case, I submitted. The sequel has proven that I was right. I reduced the fracture and dislocation in the usual manner, but it did no good. The child is still in the same condition; no union has occurred.

The history of the case, as given by the mother, is, that six or eight weeks previous to her confinement she attempted to jump from a cart while the oxen were running, and she fell upon her stomach. She felt the child give an unusual hard jump, as she expressed it, immediately after the fall.

I would respectfully ask the editors of the JOURNAL if my proposed mode of operation was correct?

Respectfully, JULIAN T. MOORE, M.D. (?)

Fractures in utero are to us novel. We publish the above rare accident, and would say to our correspondent that if soft structures intervene, the fractured ends of bone cannot, of course, unite until they are removed. A comminuted fracture, however, may fail to become firm, owing to disturbance of nutrition in the smaller pieces of bone. These may not only fail to throw out callus, but, intervening, prevent the union of still vital portions of the shaft.

If skin or muscle intervene, manipulation would seem to be sufficient to insure co-adaptation of the fractured ends, and time will determine the existence of dead portions of bone, by the establishment of the suppurative process, and formation of abscesses.—[Ed.]

